# Synergistic effect of CD47 blockade in combination with cordycepin treatment against cancer

**DOI:** 10.3389/fphar.2023.1144330

**Published:** 2023-04-17

**Authors:** Chen Feng, Rongzhang Chen, Weiwei Fang, Xinran Gao, Hanjie Ying, Xiao Zheng, Lujun Chen, Jingting Jiang

**Affiliations:** ^1^ Department of Tumor Biological Treatment, The Third Affiliated Hospital of Soochow University, Chang Zhou, Jiang Su, China; ^2^ Jiangsu Engineering Research Center for Tumor Immunotherapy, The Third Affiliated Hospital of Soochow University, Chang Zhou, Jiang Su, China; ^3^ Institute of Cell Therapy, The Third Affiliated Hospital of Soochow University, Chang Zhou, Jiang Su, China; ^4^ College of Biotechnology and Pharmaceutical Engineering, Nanjing Tech University, Nanjing, Jiang Su, China

**Keywords:** cordycepin, anti-CD47, macrophage, tumor microenvironment, scRNA-seq

## Abstract

Cordycepin is widely considered a direct tumor-suppressive agent. However, few studies have investigated as the effect of cordycepin therapy on the tumor microenvironment (TME). In our present study, we demonstrated that cordycepin could weaken the function of M1-like macrophages in the TME and also contribute to macrophage polarization toward the M2 phenotype. Herein, we established a combined therapeutic strategy combining cordycepin and an anti-CD47 antibody. By using single-cell RNA sequencing (scRNA-seq), we showed that the combination treatment could significantly enhance the effect of cordycepin, which would reactivate macrophages and reverse macrophage polarization. In addition, the combination treatment could regulate the proportion of CD8^+^ T cells to prolong the progression-free survival (PFS) of patients with digestive tract malignancies. Finally, flow cytometry validated the changes in the proportions of tumor-associated macrophages (TAMs) and tumor-infiltrating lymphocytes (TILs). Collectively, our findings suggested that the combination treatment of cordycepin and the anti-CD47 antibody could significantly enhance tumor suppression, increase the proportion of M1 macrophages, and decrease the proportion of M2 macrophages. In addition, the PFS in patients with digestive tract malignancies would be prolonged by regulating CD8^**+**^ T cells.

## Introduction

Cordycepin (3′-deoxyadenosine) is a critical bioactive component of *Cordyceps militaris*, which has a wide range of biological effects, such as antitumor, anti-inflammatory, and antidiabetic properties. Recent experiments have shown that cordycepin has a direct and safe antitumor efficacy in breast cancer (Liu et al., 2020a), cholangiocarcinoma (CCA) (Wang et al., 2017; Liu et al., 2020b), non-small cell lung cancer (NSCLC) (Wei et al., 2019), and bladder cancer (Kim et al., 2019). Furthermore, accumulating evidence has indicated that cordycepin can attack the DNA, induce the production of reactive oxygen species (ROS), and promote apoptosis by deactivating the PI3K/AKT pathway (Nasser et al., 2017; Zeng et al., 2017; Kim et al., 2019; Wang et al., 2019). In addition, cordycepin can enhance cisplatin and temozolomide sensitivity by inhibiting the PI3K/AKT pathway (Bi et al., 2018; Gao et al., 2020). Moreover, with regard to breast cancer, multiple studies have shown that cordycepin can suppress epithelial–mesenchymal transition (EMT), invasion, and metastasis by inhibiting the Hedgehog pathway and the generation of ROS (Liu et al., 2020a; Wei et al., 2022). Overall, cordycepin inhibits migration, proliferation, and invasion of tumor cells and EMT *via* promoting apoptosis. In addition, cordycepin can significantly suppress inflammatory responses in chronic kidney disease (Sun et al., 2019), acute pancreatitis (Yang et al., 2020), and acute pneumonia (Kim et al., 2011) by inhibiting both the phosphorylation of MAPKs and the activation of NF-κB.

In recent years, immunotherapy has emerged as an essential treatment for cancer, in addition to surgery, chemotherapy, and radiotherapy. CD47 is a cell surface molecule that is widely expressed in most cancers. It consists of an extracellular domain known as IgSF, five transmembrane domains, and one variable intracellular domain. Accumulating evidence showed a positive connection between high CD47 expression and poor prognoses in various cancers, such as NSCLC (Arrieta et al., 2020) and gastric cancer (GC) (Shi et al., 2021). The SIRP family includes five primary members, namely, SIRPα, SIRPβ1, SIRPβ2, SIRPγ, and SIRPδ. Among them, SIRPα and SIRPγ, when activated by their corresponding CD47 receptors, played an inhibitory role. When CD47 interacts with SIRPα, the immune-receptor tyrosine-based inhibitory motif (ITIM), the intracellular domain of SIRPα is activated and phosphorylated. In addition, the phosphatases SHP-1 and SHP-2 are activated by ITIM (Kharitonenkov et al., 1997; Jiang et al., 1999). The heterogeneity of macrophages has different responses to pathogens and external stimuli. Macrophages have been activated in a kind of continuous mode from classically activated (M1) macrophages (pro-inflammatory/antitumor) to activated (M2) macrophages (anti-inflammatory/pro-tumor) (Goerdt et al., 1999; Mantovani et al., 2002; Sica et al., 2008). Increased tumor-associated macrophage (TAM) infiltration and phagocytic function are found when the interaction between CD47 and SIRPα is blocked (Weiskopf et al., 2016; Li et al., 2018; Schürch et al., 2019). In addition, it has been confirmed that the blockade of the CD47-SIRPα pathway in the treatment of tumors can significantly increase the phagocytic function of M1 macrophages, leading to a converse shift from M2 macrophages to M1 macrophages (Zhang et al., 2016). Two clinical trials (EudraCT numbers: 2016-004372-22 and NCT02641002) were terminated in 2017 and 2018 due to severe anemia that resulted in adverse drug reactions (ADRs). However, in 2019, a clinical trial about the combination treatment of the CD47 antibody and azacytidine for acute myeloid leukemia (AML) and myelodysplastic syndrome (MDS) indicated high treatment efficacy and high ADRs (Sallman et al., 2019).

In this study, we investigated the effectiveness of a combination treatment using an anti-CD47 antibody and cordycepin to enhance tumor inhibition. By using single-cell RNA sequencing (scRNA-seq), we confirmed that this combination treatment increased the proportion of M1-like macrophages and decreased the proportion of M2-like macrophages. Furthermore, the combination treatment was found to prolong progression-free survival (PFS) in patients with colorectal cancer (CRC) by regulating CD8^+^ T cells. We also used flow cytometry to analyze the reshaping of the tumor microenvironment (TME). In conclusion, this combination treatment proved to be a valuable therapeutic strategy for various types of cancer.

## Materials and methods

### Cell culture and experimental animals

The mouse colon cancer cell lines MC38 and CT26 were provided by the Chinese Academy of Sciences, Shanghai Institutes for Biological Sciences (Shanghai, China). Briefly, MC38 and CT26 cells were maintained in DMEM (Gibco, Thermo Fisher Scientific, United States) supplemented with 10% (v/v) fetal bovine serum (FBS) and 100 U/mL penicillin. In addition, male C57BL/6J and Balb/c mice (6–8 weeks old) were housed in a specific pathogen-free (SPF) environment in Cavens Laboratory Animals (Jiangsu Changzhou, China). All animal-related procedures were approved by the Ethics Committee of the Third Affiliated Hospital of Soochow University.

### Animal models and *in vivo* treatment

Briefly, 1 × 10^6^ MC38 cells were injected under the skin (s.c.) on the right flank of C57BL/6J mice, and 1e^−6^ CT26 cells were injected subcutaneously (s.c.) on the right flank of Balb/c mice. Different treatments were given when the maximal diameter of the tumor was greater than 5 mm, including PBS, cordycepin, CD47 antibody (Clone MIAP301, BioXcell, United States), and combination therapy (anti-CD47+ cordycepin) groups. The mice were treated with the CD47 antibody (200 µg/mouse 200 *i. p.*) on days 0, 4, 8, and 12 during the treatment. Extraction and purification of cordycepin were completed by the College of Biotechnology and Pharmaceutical Engineering, Nanjing Tech University. Cordycepin was kept in clean and dried tubes at 4°C. It was dissolved in saline. The mice were treated daily with cordycepin (500 µg/mouse, i. g.). The tumor volume was measured every 2 days and calculated using the formula as follows: V = Length × Width^2^ × π/6.

### Flow cytometry

The tumor tissues were minced into pieces smaller than 1 mm^3^. Next, the tumors were digested with DNase I (REF 10104159001, Roche) and Liberase TL (REF 05401020001, Roche). Once the digestion was complete, a serum-containing culture medium was added, followed by grinding and screening through a 200-µm strainer to obtain a single-cell suspension. Antibodies against mouse CD45 (Clone 30-F11, BD Biosciences), Ghost (No. 59863S, Cell Signaling Technology), CD3 (Clone 17A2, BD Biosciences), CD4 (Clone GK1.5, BD Biosciences), CD8 (Clone 53-6.7, BD Biosciences), GR1 (Clone RB6-8C5, BD Biosciences), CD11b (Clone M1/70, BD Biosciences), MHC-II (Clone M5/114.15.2, BD Biosciences), F4/80 (Clone BM8, BD Biosciences), and CD206 (Clone CO68C2, BD Biosciences) were used to stain cells. Data acquisition was conducted using a Beckman Coulter DxFLEX cytometer, followed by data analysis using FlowJo software (version 10.8.1, FlowJo Software, United States).

### scRNA-seq

Using the aforementioned method, single-cell suspensions of the tumors were prepared. For FACS sorting, the cells were enriched using the CD45 [tumor-infiltrating lymphocytes (TILs)] Microbead Mouse Kit (Cat. 130-110-618, Miltenyi Biotec, Lerden, Netherlands) and stained with Ghost Dye™ Violet 510 Viability Dye (No. 59863S, Cell Signaling Technology) and Percp-Cy5.5-CD45 (Clone 30-F11, BD Biosciences) antibodies. Each sample contained approximately 1 × 10^6^ CD45^+^ cells sorted using the BD FACSAria II instrument. FACS analysis was used to sort single cells into flow tubes, and the AOPI was used to determine the viability of the cells. In order to produce single-cell gel beads in the emulsion, the cell suspension, which contained 300–600 living cells per microliter according to CountStar, was loaded onto the chromium single-cell controller (10x Genomics). Using an S1000TM Touch Thermal Cycler (Bio-Rad), single-cell transcriptome amplification was conducted at 53°C for 45 min, followed by incubation at 85°C for 5 min and holding at 4°C for 10 min. An Agilent 4200 instrument was used to assess the quality of the cDNA templates (performed by CapitalBio Technology, Beijing).

### scRNA-seq data processing

Gene-barcode matrices were generated using Cell Ranger (v5.0.0) software by demultiplexing and aligning raw sequencing data to the mm10 mouse reference genome. Filtration, normalization, dimensionality reduction, clustering, and differential expression analysis were performed using the Seurat (v4.1.1) R package. The following criteria were applied to remove low-quality cells: gene number between 200 and 6,000, UMI count >1,000, and mitochondrial content >10%. A total of 12,907 cells were obtained after filtration. The batch effect across different samples was eliminated using the Harmony (v0.1.0) method. The “ElbowPlot” function was adopted to select the top 40 harmony embeddings to perform clustering and visualization. Different clustering results were generated using the “FindClusters” function with resolutions ranging from 0.2 to 1.2. A suitable resolution was determined using the clustree package (v0.5.0) in R. Finally, 24 clusters were obtained (resolution = 1.2). A total of 12,772 cells were retained for further analysis after excluding two clusters (18) that did not express any known markers. The MAST method (“FindAllMarkers”) was used for differential gene analysis. Log2-fold changes greater than 0.25 and Bonferroni-adjusted *p*-values less than 0.05 were used to identify differentially expressed genes (DEGs). In the second round of clustering for CD8^+^ T cells and myeloid cells, the SCTransform method was used to normalize the expression matrix, the Harmony method was used for integration analysis, and the FindNeighbor and FindCluster functions were used for clustering.

### Gene Expression Omnibus (GEO) data

The GEO dataset was downloaded from the GEO database (http://www.ncbi.nlm.nih.gov/geo)(GEO accession No. GSE126157). Principal component analysis (PCA) was conducted using the FactoMineR and factoextra R packages. DEGs were identified using the limma R package. Heatmap was constructed using the limma, ggplot2, and pheatmap packages. Finally, KEGG (Kyoto Encyclopedia of Genes and Genomes) pathway analysis was carried out using the org.Mm.eg.db and dplyr R packages.

### The Cancer Genome Atlas (TCGA) data and COX survival model

RNA-seq profiles and corresponding clinical information on CRC, CCA, GC, and esophageal carcinoma (ESCC) were downloaded from TCGA database (https://portal.gdc.com). The count data were converted to TPM and normalized, matching with the corresponding clinical information. Log-rank was used to compare differences in survival. The timeROC (version 0.4) analysis was used to compare the predictive accuracy. R package survival was used to build the COX survival model. For Kaplan–Meier (KM) curves, *p*-values and hazard ratios (HRs) with 95% confidence interval (CI) were generated by log-rank tests and univariate cox proportional hazards regression. HOME for researchers (www.home-for-researchers.com) was used to analyze the TCGA data.

### Statistical analysis

GraphPad Prism V.10 software was used to perform statistical analyses, and the data were expressed as mean ± SEM. The one-tailed, unpaired Student’s t-test was used to compare the two groups. In addition, tumor growth curves were compared by the two-way ANOVA. Finally, the survival of the mice was analyzed using the KM method and the log-rank test. *p* < 0.05 was considered statistically significant.

## Results

### Anti-CD47 antibody in combination with cordycepin shows a significant therapeutic effect on colon cancer models in mice

**FIGURE 1:**
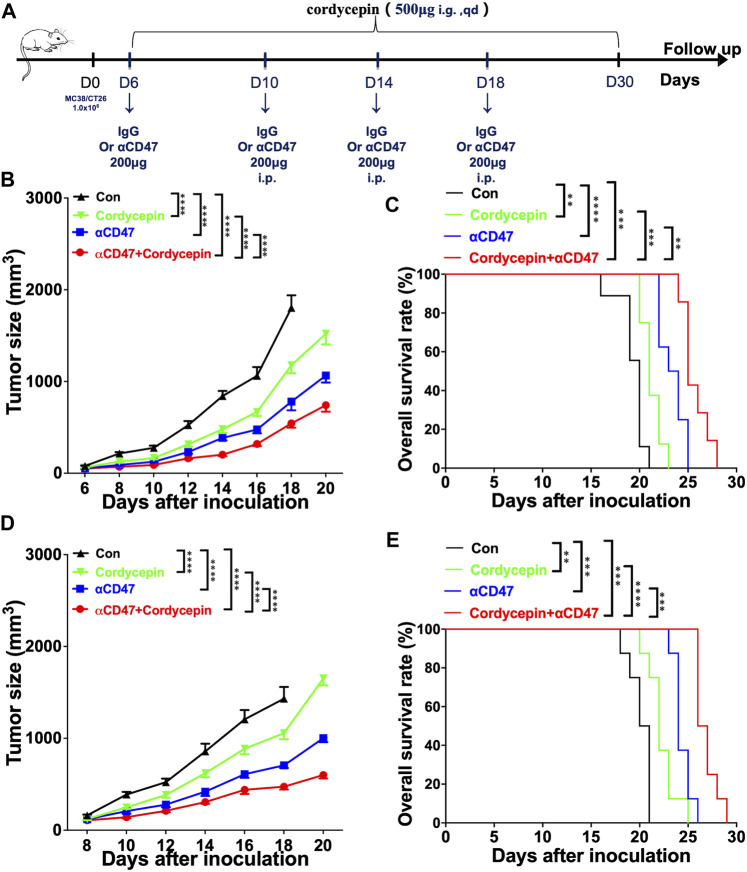
Combination treatment shows significant therapeutic effects in animal models **(A)**. Schematic illustration of the experimental design time flow. MC38 tumor-bearing mice or CT26 tumor-bearing mice were treated with anti-CD47 antibodies or IgG (control) on days 6, 10, 14, and 18 after tumor inoculation. The mice were treated with cordycepin *via* daily gavage **(B, D)**. Tumor growth curves of all groups, following the injection of 1 × 10^6^ MC38 or CT26 cells (*n* > = 6, representative results from three independent experiments) **(C, E)**. KM survival analysis of the mice from **(B, D)** (log-rank (Mantel–Cox) test) (*n* = 6, representative results from three independent experiments). n. s. (not significant) *p* > 0.05, ^*^ *p* < 0.05, ^**^ *p* < 0.01, ^***^ *p* < 0.001, and ^****^ *p* < 0.0001.

We constructed the subcutaneous tumor models to validate the combined therapeutic effect using the murine CRC cell lines MC38 and CT26. Then, the tumor-bearing mice were subjected to anti-CD47, cordycepin, and combination treatments. Cordycepin was administered daily by oral gavage for 30 days. In addition, the anti-CD47 antibody and control IgG were administered every 4 days *via* intraperitoneal injection (Figure 1A). For mouse models of MC38 CRC, combination treatment significantly decreased the proliferation of tumor cells and prolonged survival (Figures 1B, C). Moreover, for mouse models of CT26, the combination treatment showed a significant effect. Taken together, the combination treatment decreased the proliferation of tumor cells and prolonged survival (Figures 1D, E).

### Cordycepin inhibits the function of M1 macrophages

**FIGURE 2:**
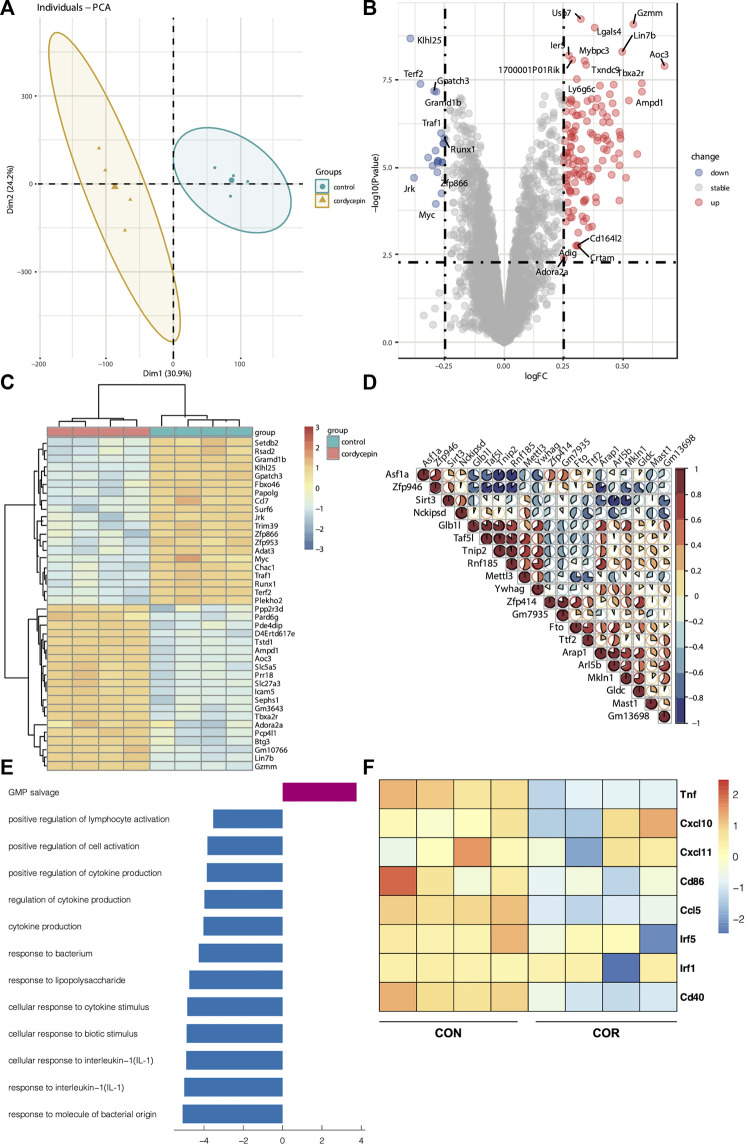
Cordycepin decreases the function of macrophage I (M1 macrophages) **(A)**. PCA showed the difference between the two groups **(B)**. DEGs of two groups. The marked genes by tags were those with a *p*-value < 0.005 and a log Fc > 0.25 **(C)**. Heatmap was chosen to show the top 20 up- and downregulated genes **(D)**. Heatmap was chosen to show the correlations of the top 20 genes **(E)**. KEGG pathway enrichment analysis was performed utilizing the KEGG pathway database (http://www.genome.jp/kegg). Based on the function of macrophages, the pathways were selected. The decreasing function is represented by the bar graphs (blue), and the increasing function is represented by the bar graphs (red) **(F)**. Heatmap was chosen to show the differential gene expression of molecular markers of the M1 phenotype. Cordycepin was represented by “COR”.

Many studies have confirmed that cordycepin exerts its antitumor effects directly by inducing the apoptosis of tumor cells (Wang et al., 2017; Jin et al., 2018; Liu et al., 2020a). To test the effect of cordycepin on macrophages, we found the matrix used to record the gene expression of macrophages treated by cordycepin (GSE126157) (Ashraf et al., 2019). We selected the LPS-induced macrophage group and LPS-induced macrophages treated with the cordycepin group as a new matrix. This new matrix was then subjected to PCA. Moreover, we found that PCA could discriminate between the control and cordycepin groups (Figure 2A), indicating that cordycepin could significantly influence the function of M1-like macrophages. DEG analysis showed that the expressions of the *Myc* and *Ccl7* genes were decreased in the cordycepin group (Figure 2B), confirming that the *Myc* gene could promote macrophage proliferation in a non-inflammatory manner (Yin et al., 2020; Gerlach et al., 2021). In addition, the *Ccl7* gene could promote macrophage polarization to M1-like macrophages and increase the level of macrophage infiltration (He et al., 2019; Xie et al., 2021). We found that the regulatory genes were enriched in multiple pathways (Figures 2C, D). In addition, enrichment and signaling pathway analysis were performed for DEGs (Figure 2E). We found that cordycepin could dampen the function of M1-like macrophages in the LPS-induced macrophage model (Figure 2E), such as by decreasing the interaction between macrophages and IL-1 and cytokine production. Finally, gene markers of the M1 macrophages were compared in DEGs, and we found that cordycepin would decrease the expression of these molecular markers (Figure 2F).

### Anti-CD47 reverses the inhibitory effect of cordycepin on M1-like macrophages

We found that the combination treatment resulted in better effects than the cordycepin treatment alone (Figure 1), and the cordycepin treatment could weaken the function of M1-like macrophages (Figure 2). Therefore, we hypothesized that the anti-CD47 antibody would increase the inhibitory effect. To test this hypothesis, we used scRNA-seq in MC38 tumor-bearing mice. Starting from the second day of the third anti-CD47 mAb treatment cycle, cordycepin was administered daily through gavage. The tumors were dissociated into a single-cell suspension. The dissociated cells were positively selected using CD45 microbeads and sorted by flow cytometry (Supplementary Figures S1A, B). Furthermore, all cells were divided into six clusters, including B cells, CD4^+^ T cells, CD8^+^ T cells, γδT, myeloid cells, and natural killer (NK) cells (Supplementary Figure S1C).

**FIGURE 3:**
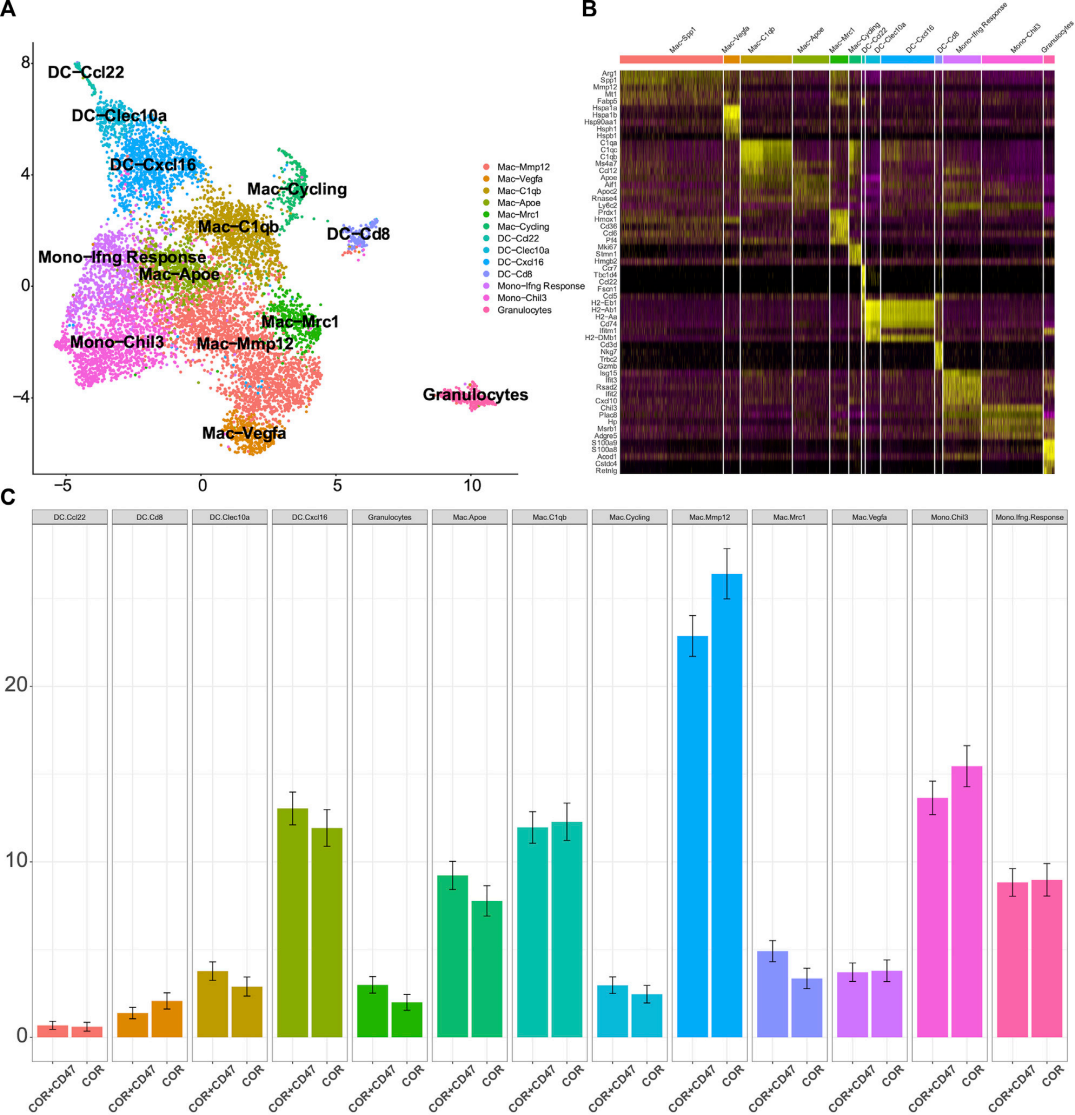
scRNA-seq is used to test the changes in myeloid cells **(A)**. Sub-clustering of myeloid cells is shown on the UMAP plots of the scRNA-seq dataset **(B)**. Individual myeloid cell clusters in the scRNA-seq data could be identified phenotypically based on a heatmap of myeloid cell lineage and functional markers **(C)**. MC38 tumors from the combination treatment group or cordycepin group showed the proportions of different subpopulations of myeloid cells. “COR + CD47” represents the combined treatment group; “COR” represents the cordycepin treatment group.

**FIGURE 4:**
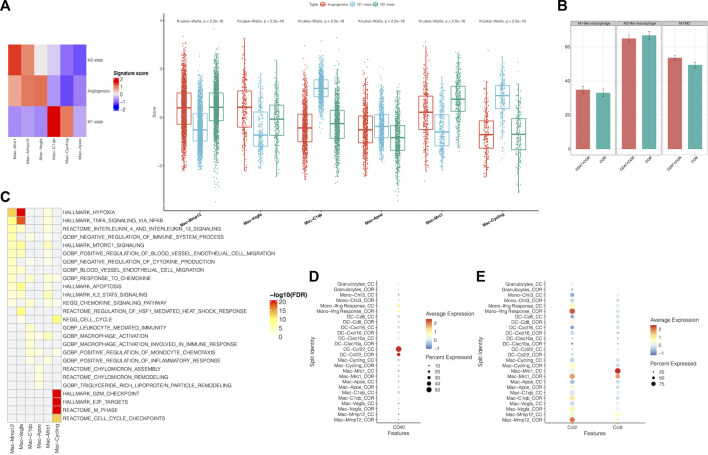
Function of sub-clustering in myeloid cells is confirmed by scRNA-seq **(A)**. Using the scRNA-seq data, M1-state, M2-state, and angiogenesis signature scores for each myeloid cell cluster are shown by a heatmap and boxplot **(B)**. This bar graph shows the changes of M1-like macrophages and M2-like macrophages **(C)**. Using the KEGG database**,** pathways were significantly enriched in the scRNA-seq data for each macrophage cluster **(D)**. A dot plot showed the expression of CD40 on myeloid cells in MC38 tumors from the combination treatment group or cordycepin group **(E)**. Different expressions of chemokines in different subpopulations of myeloid cells are shown by a dot plot in MC38 tumors from the combination treatment group or cordycepin group. “COR + CD47” represents the combined treatment group; “COR” represents the cordycepin treatment group.

The cluster of myeloid cells was chosen to analyze the function of dendritic cells (DCs) and macrophages. First, we used the DEGs to define all clusters (Figures 3A, B). We found that the number of M2 macrophages in the combination treatment group decreased, which accounted for a high proportion of all macrophages. For DCs, *Cxcl16* DCs and *Clex10a* DCs were increased in the combination treatment group (Figure 3C). Moreover, we determined the M1-like macrophage score and the M2-like macrophage score to assess the function of TAMs. Genes associated with M1 macrophages include *C1qc*, *C1qb*, *Il23*, *Tnf*, *Cxcl9*, *Cd86*, *Il1a*, *Il1b*, *Il6*, *Ccl5*, *Irf7*, *Irf1*, *Cd40*, *Ido1*, *Cx3cr1*, and *Trem2*. In addition, genes associated with M2 macrophages include *Il4r*, *Ccl4*, *Ccl13*, *Ccl20*, *Ccl17*, *Ccl18*, *Ccl24*, *Lyve1*, *Vegfa*, *Vegfb*, *Vegfc*, *Vegfd*, *Egf*, *Ctsa*, *Ctsb*, *Ctsc,* and *Tgfb1*. We observed that M2 macrophages had higher M2-like macrophage scores (Figure 4A). We used the M1-like macrophage score and the M2-like macrophage score to classify cells and identify changes in the number of macrophages. Our findings showed that cordycepin treatment increased M1-like macrophages and decreased M2-like macrophages (Figure 4B). Then, we used the KEGG database to enrich the pathway for each macrophage cluster. In addition, we found that the genes of M2 macrophages were enriched in the hypoxia pathway and TNFα signaling pathway (Figure 4C). In addition, we explored the expression of CD40 in macrophages and DCs. *CCL22* ^*−*^ DCs were found to have a higher expression of CD40 (Figure 4D), which would be a key factor for enhancing antigen presentation capacity (Bullock, 2022). Moreover, the expression of *CCL2* on macrophages and DCs was decreased, and the expression of *CCL9* was increased (Figure 4E). This finding confirmed the correlation between *CCL9* and cytotoxicity (Matloubian et al., 2000; Dangaj et al., 2019; Di Pilato et al., 2021). Furthermore, the expression of *CCL2* has been confirmed to increase tumor cell proliferation through the polarization of TAMs (McClellan et al., 2012).

We found that the combination treatment of the anti-CD47 antibody and cordycepin could decrease M2-like macrophages and increase M1-like macrophages. Furthermore, the combination treatment could enhance the antigen-presenting ability by increasing the expression of CD40. Finally, the combination treatment could regulate *CCL2* and *CCL9* to mediate the functions of macrophages.

### Anti-CD47, in combination with cordycepin, regulates CD8^+^ T-cell-mediated antitumor immune response

**FIGURE 5:**
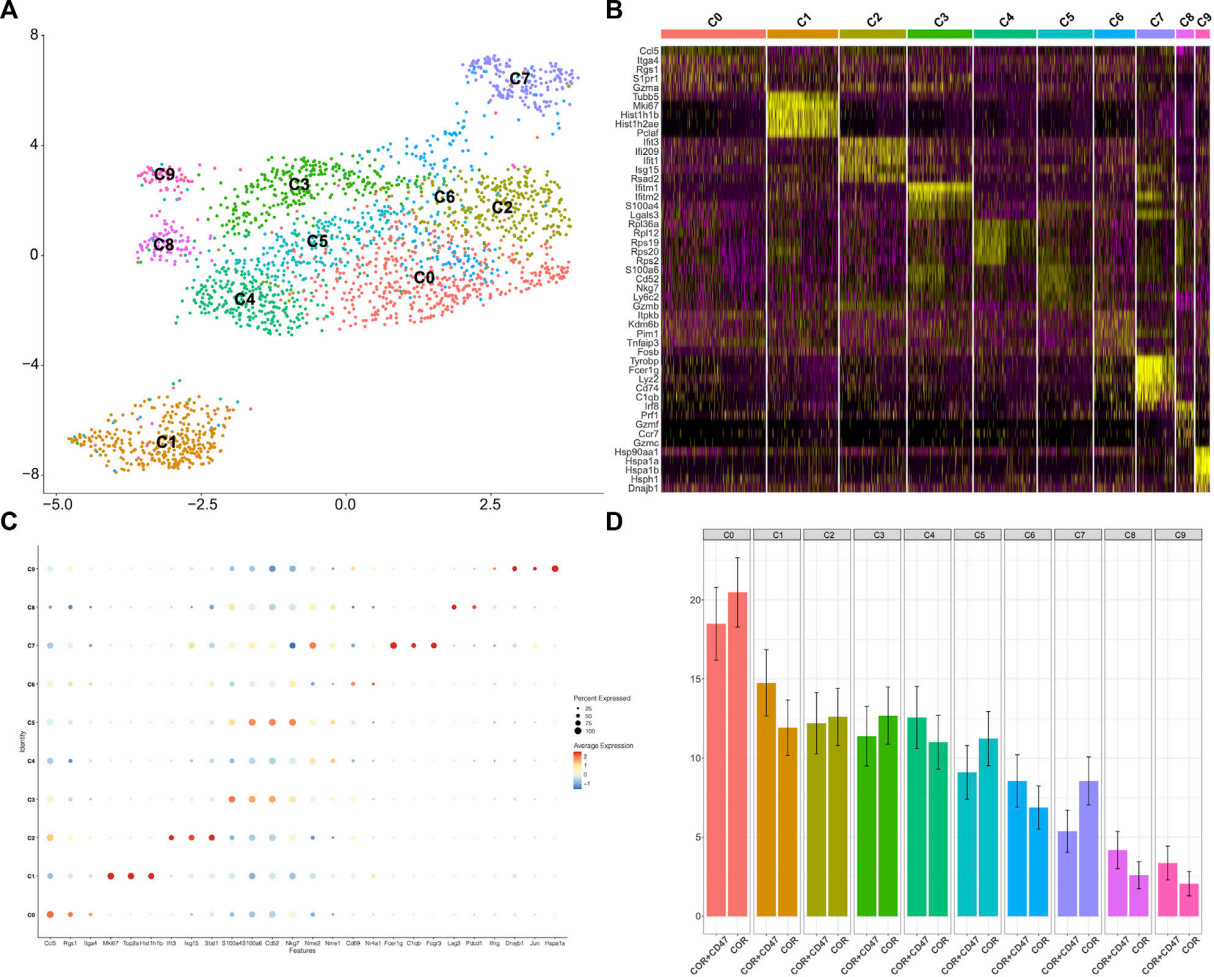
scRNA-seq analysis is used to test the changes of CD8^+^ T cells **(A)**. UMAP plots of the scRNA-seq dataset show sub-clustering of CD8^+^ T cells **(B)**. Individual CD8^+^ T-cell clusters in the scRNA-seq data could be identified phenotypically based on a heatmap of CD8^+^ T-cell lineage and functional markers **(C)**. A dot plot showed the expression levels and activity scores of known phenotypic markers in each CD8^+^ T-cell cluster **(D)**. The proportions of different subpopulations of CD8^+^ T cells are shown by bar graphs in MC38 tumors from the combination treatment group or cordycepin group.

**FIGURE 6:**
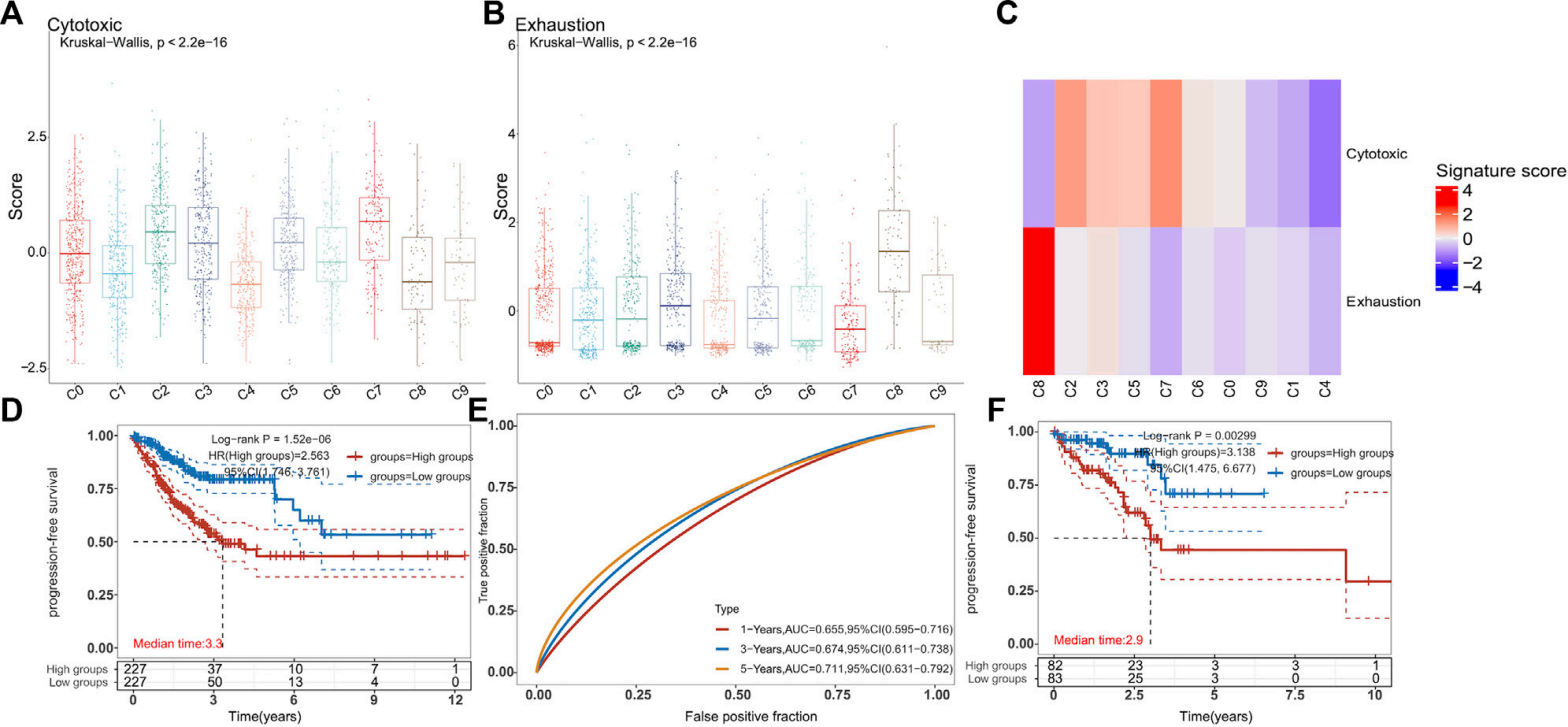
Combination treatment is confirmed to increase the PFS of patients with digestive tract malignancy by regulating the CD8^+^ T-cell sub-cluster **(A–C)**. In the scRNA-seq data, boxplots and heatmaps showed the cytotoxicity and exhaustion signature scores for each CD8^+^ T-cell cluster. The VISION method was used to calculate signature scores for each cell cluster (two-sided Wilcoxon test) **(D)**. KM survival curves of TCGA-CRC patients (*n* = 455) grouped by the averaged expression of the COX model (with the median value as the threshold). HR (high expression) represents the hazard ratio of the low-expression sample relative to the high-expression sample. HR (95% Cl) represents the median survival time (LT50) for different groups. The blue line represents the low expression of the average expression of the COX model, and the red line represents the high expression **(E)**. The ROC model was used to test the accurate survival prediction for 1 year, 3 years, and 5 years. The higher AUC score corresponded to higher predictive power **(F)**. The rectal cancer data and clinical information were downloaded from TCGA database (*n* = 165). KM survival curves **(F)** were the same as **(E)**.

Because of the increased expression of CD40 in myeloid cells in the combination treatment group, we further explored the proportions of various T-cell subsets. The scRNA-seq data on CD8^+^ T cells were chosen to reduce the dimension of features. Moreover, the DEGs were used to discriminate 10 clusters (Figure 5A). The C0 cluster represented a majority of CD8^+^ T cells, which exhibited high levels of *CCL5*, *Rgs1*, and *Itga4* (Figures 5B, C). In addition, the combination treatment decreased these clusters (Figure 5D). In contrast, the combination treatment increased the C1 cluster, which showed high expressions of *Mki67*, *Top2a*, and *Hist1h1b*. To explore the function of all clusters, we determined the exhaust score and cytotoxic score for all clusters. We found that the C0 cluster and the C1 cluster had lower exhaustion (Figures 6A, B). In addition, the C8 cluster got the highest exhaust score (Figure 6C). Because the C0 cluster accounted for a significant proportion among all clusters, we used TCGA database to test the function of the C0 cluster for PFS in digestive tract malignancy. We set up a COX survival model using the DEGs of the C0 cluster (Supplementary Figure S2A). The survival data on CRC were chosen to test the COX model. We found that the low-level group showed better PFS (Figure 6D). To evaluate survival prediction, we found that the 5-year AUC of the COX model was 0.711 (Figure 6E), indicating that the COX survival was an accurate prediction model for the 5-year PFS of CRC. Carcinoma data from the TCGA were used to test the COX model. It was confirmed that patients in the low-score group had better PFS (Figure 6F). GC and ESCC data from the TCGA were used to test the COX model. We found that the PFS for GC and ESCC might be prolonged using the combination treatment through regulating CD8^+^ T-cell subsets (Supplementary Figures S2B, C). Therefore, we confirmed that the combination treatment would regulate CD8^+^ T-cell subsets, which could decrease the C0 cluster to improve PFS for digestive tract malignancy.

### Combination treatment changes the proportion of TAMs

**FIGURE 7:**
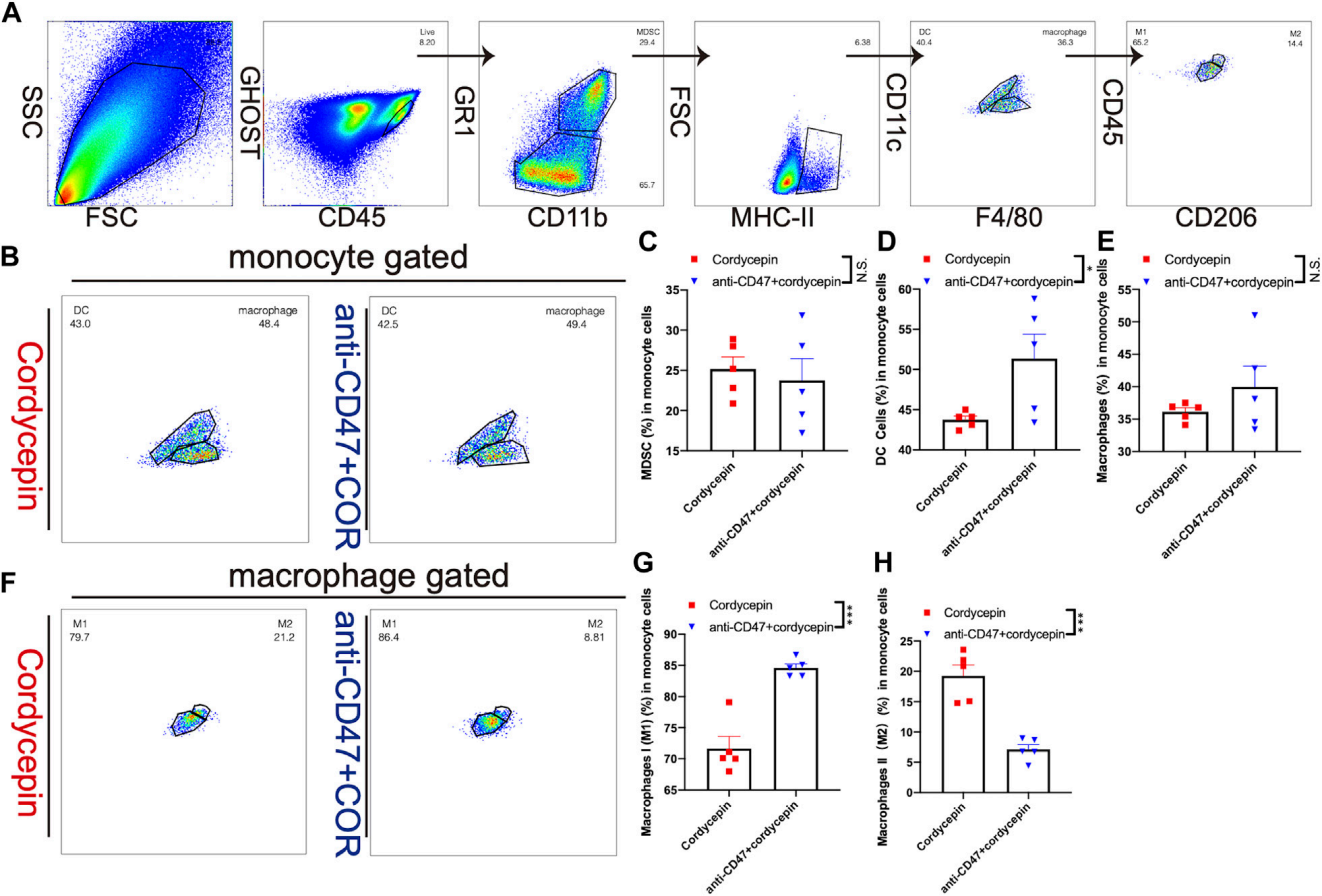
Change level of TAMs is shown by flow cytometry **(A)**. Gating strategy for flow cytometric phenotyping was used to identify the proportions of macrophages and DCs **(B)**. Different proportions of macrophages and DCs are shown by a dot plot. (*n* = 5, representative results from three independent experiments) **(C–E)**. Bar graphs show a difference in the proportions of MDSCs, macrophages, and DCs. The error bars showed SEM representative results from three independent experiments **(F)**. Different proportions of M1-like and M2-like macrophages (*n* = 5, representative results from three independent experiments) **(G, H)**. Bar graphs show different proportions of M1-like and M2-like macrophages. Error bars show SEM representative results from three independent experiments. n. s. (not significant) *p* > 0.05, ^*^ *p* < 0.05, ^**^ *p* < 0.01, ^***^ *p* < 0.001, and ^****^ *p* < 0.0001.

We used flow cytometry to test the changes in TILs and TAMs in MC38 tumor-bearing mice. We explored the effect of the combination treatment on myeloid cells. First, macrophages were gated as MHC-II and F4/80, and DCs were gated as MHC-II and CD11c. Moreover, we used the expression of CD206 to distinguish M1-like and M2-like macrophages (Figure 7A). We found that the proportion of myeloid-derived suppressor cells (MDSCs) was not significantly changed (Figure 7C). Therefore, it was confirmed that the combination treatment increased the proportion of DCs (Figures 7B, D). In addition, the proportion of macrophages was not significantly changed (Figures 7B, E). However, the proportions of macrophage subsets were adjusted: the number of M1-like macrophages was increased, and the number of M2-like macrophages was decreased (Figures 7F–H). In addition, the proportion of TILs was confirmed by flow cytometry, showing that the proportions of CD3^+^ T cells, CD4^+^ T cells, and CD8^+^ T cells were not significantly changed (Supplementary Figures S3A–D). These findings suggested that the combination treatment could regulate CD8^+^ T-cell subsets rather than increase the proportion of CD8^+^ T cells.

## Discussion

In the present study, we examined the effect and explored the mechanism of the combination treatment of the anti-CD47 antibody and cordycepin. Cordycepin has been well known as an effective tumor inhibitor, while its inhibitory effect on macrophages can also limit its antitumor effect. Our current study aimed to reveal whether the combination treatment had a remarkable effect against MC38 and CT26 tumor-bearing mouse models. Using the scRNA-seq technique, we confirmed that the combination treatment increased the proportion of M1-like macrophages and decreased the proportion of M2-like macrophages. In addition, the combination treatment could increase the CD8^+^ T subset to improve the survival of patients with digestive tract malignancies. Finally, flow cytometry showed that the combination treatment could increase the proportion of M1 macrophages and decrease the proportion of M2 macrophages.

In Chinese traditional medicine, *Cordyceps militaris* has been confirmed as an essential medicine dealing with fever, hemoptysis, and tumors (Hsu et al., 2008; Das et al., 2020). Cordycepin is the potent, effective constituent of *Cordyceps militaris*. It is widely accepted to be a direct antitumor inhibitor. Cordycepin is improved to decrease the drug resistance of NSCLC by inhibiting the AMPK signaling pathway and inhibit NSCLC with cisplatin resistance by the AMPK signaling pathway and AKT signaling pathway (Wei et al., 2019) (Liao et al., 2020). Moreover, a combination of cordycepin and apatinib can suppress the progression of NSCLC *via* the VEGF/PI3K/AKT signaling pathway (Wei et al., 2019). For breast cancer, cordycepin is confirmed as an essential inhibitor of the Hedgehog pathway, which can regulate angiogenesis, aggressive molecular subtypes, and the metastatic potential of this malignancy (Liu et al., 2020a; Wu et al., 2022) (Di Mauro et al., 2017; Riaz et al., 2018). Because of the high correlation between breast cancer and hormone secretion, cordycepin also serves as a direct inhibitor for ER-independent breast cancer (Choi et al., 2011). Cordycepin has been shown to be a direct inhibitor in many studies of CRC (Lee et al., 2013; Deng et al., 2022). In addition to the direct killing effect of cordycepin, the effect of cordycepin on the TME should be considered. It has been reported that cordycepin is an excellent inhibitor of acute inflammation (Lan et al., 2021; Wei et al., 2021; Chen et al., 2022). Studies have shown that cordycepin can increase the polarization of M2-like macrophages and downregulate M1-like macrophages in sepsis mouse models (Chen et al., 2022). In Alzheimer’s disease, cordycepin has been found to enhance the polarization of microglia from M1 to M2 (Wei et al., 2021). Cordycepin has also been observed to decrease macrophage function and increase M2 polarization in multiple models of inflammation. Although cordycepin has a significant effect on suppressing tumor growth, its suppressive effect on macrophages limits its antitumor activity.

Blockade of CD47 signaling is a recently developed regimen targeting macrophages and DCs (Tahk et al., 2021; Cao et al., 2022). Targeting the CD47/SIRP signaling pathway can increase the antibody-dependent cellular phagocytosis (ADCP) of macrophages and regulate macrophage polarization (Kulkarni et al., 2018; Chen et al., 2019). For melanoma, breast cancer, and NSCLC, blocking the CD47/SIRPα pathway is an effective treatment strategy (Zhang et al., 2017; Hu et al., 2020; Rao et al., 2020). Moreover, sorafenib, in combination with anti-CD38 and anti-GD2 antibodies, can significantly increase its effect on inhibiting the tumor (Huang et al., 2021; Müller et al., 2022; Theruvath et al., 2022). However, because of the broad expression of CD47, an essential mechanism of immune regulation is the phagocytosis of old RBCs by macrophages. In clinical trials, anemia is the most common adverse effect of blocking the CD47/SIRPα pathway (Advani et al., 2018; Harrison et al., 2022). Furthermore, it has been confirmed that cordycepin can decrease the expression of CD47 on tumors in CRC (Deng et al., 2022). However, considering the inhibitory effect of cordycepin on macrophages in the TME, we did not believe that the inhibitory effect of CD47 expression would stimulate the ADCP. Notably, based on Chinese medicine theory, the regulation of cordycepin is bidirectional. Therefore, long-term cordycepin treatment could enhance immune cell function. Generally, we explored the combination treatment of cordycepin and anti-CD47 antibodies in the present study. The combination treatment could decrease the inhibitory effect of ADCP and increase the survival of CRC patients by regulating CD8^+^ T cells.

## Data Availability

The data presented in the study are deposited in the GEO repository, accession number “GSE224945”.
