# Supplementary material for: Synergistic effect of CD47 blockade in combination with cordycepin treatment against cancer
Source: Front Pharmacol. 2023 Apr 17;14:1144330. doi: 10.3389/fphar.2023.1144330 (PMC10149837; doi:10.3389/fphar.2023.1144330)
Supplement: Supplementary file 1 [file DataSheet1.docx]

Supplementary Material

**Synergistic effect of CD47 blockade in combination with cordycepin treatment against cancer**

**Chen Feng****^1,2,3†^, Rongzhang Chen^1,2,3†^, Weiwei Fang^1,2,3^, Xinran Gao^1,2,3^, Hanjie Ying**^4^**, Xiao Zheng**^1,2,3^**, Lujun Chen^1,2,3*^ and** **Jingting Jiang^1,2,3*^**

**Correspondence:** Corresponding Author:

Lujun Chen E-mail: chenlujun@suda.edu.cn

Jingtin Jiang E-mail: jiangjingting@suda.edu.cn

## Supplementary Figures

**Supplement Figure 1. A.** Process was shown the combined treatment for MC38-bearing mice model in single cell RNA sequencing. **B.**  process was shown the flow of sample preparation for single cell RNA sequencing. **C.** The dot plot was shown the clustering of cells on the UMAP plots of the scRNA-seq dataset.

**Supplement Figure 2. A.** The differential gene of C0 cluster were used to set up a cox survival model. The upper dot plot was used to represent risk score from low to high and different colors represent different groups. The middle dot plot was used to show the time level responding survival status. The lower dot heatmap represents the expression level of COX model. **B.** The Gastric cancer data from TCGA was used to test the COX model. **C.** The esophageal carcinoma data from TCGA was used to test the COX model.

**Supplement Figure 3. The change percentage of TIL was tested by** **flow cytometry. A.** Gating strategy for flow cytometric phenotyping of TIL. d (n=5 Representative results from three independent experiments.) **B**, **C** and **D.** Bar graphs were shown a difference in the percentage of CD3^+^T cells, CD4^+^ T cells, and CD8^+^ T cells. And error bars show S.E.M. Representative results from three independent experiments. n.s. (not significant) *P*>0.05, ^*^*P*<0.05, ^**^*P*<0.01, ^***^*P*<0.001, and ^****^*P*<0.0001.
